# A Century of Brain Regeneration Phenomena and Neuromorphological Research Advances, 1890s–1990s—Examining the Practical Implications of Theory Dynamics in Modern Biomedicine

**DOI:** 10.3389/fcell.2021.787632

**Published:** 2022-01-06

**Authors:** Frank W. Stahnisch

**Affiliations:** ^1^ Department of Community Health Sciences, University of Calgary, Calgary, AB, Canada; ^2^ Department of History, University of Calgary, Calgary, AB, Canada; ^3^ Hotchkiss Brain Institute, Cumming School of Medicine, University of Calgary, Calgary, AB, Canada; ^4^ O'Brien Institute for Public Health, Cumming School of Medicine, University of Calgary, Calgary, AB, Canada

**Keywords:** brain research, Ludwik Fleck, history of neuroscience, regeneration, 20th cent. history of medicine

## Abstract

The modern thesis regarding the “structural plastic” properties of the brain, as reactions to injuries, to tissue damage, and to degenerative cell apoptosis, can hardly be seen as expendable in clinical neurology and its allied disciplines (including internal medicine, psychiatry, neurosurgery, radiology, etc.). It extends for instance to wider research areas of clinical physiology and neuropsychology which almost one hundred years ago had been described as a critically important area for the brain sciences and psychology alike. Yet the mounting evidence concerning the range of structural neuroplastic phenomena beyond the significant early 3 years of childhood has shown that there is a progressive building up and refining of neural circuits in adaptation to the surrounding environment. This review essay explores the history behind multiple biological phenomena that were studied and became theoretically connected with the thesis of brain regeneration from Santiago Ramón y Cajal’s pioneering work since the 1890s to the beginning of the American “Decade of the Brain” in the 1990s. It particularly analyzes the neuroanatomical perspectives on the adaptive capacities of the Central Nervous System (CNS) as well as model-like phenomena in the Peripheral Nervous System (PNS), which were seen as displaying major central regenerative processes. Structural plastic phenomena have assumed large implications for the burgeoning field of regenerative or restorative medicine, while they also pose significant epistemological challenges for related experimental and theoretical research endeavors. Hereafter, early historical research precursors are examined, which investigated brain regeneration phenomena in non-vertebrates at the beginning of the 20th century, such as in light microscopic studies and later in electron microscopic findings that substantiated the presence of structural neuroplastic phenomena in higher cortical substrates. Furthermore, Experimental physiological research in hippocampal *in vivo* models of regeneration further confirmed and corroborated clinical physiological views, according to which “structural plasticity” could be interpreted as a positive regenerative CNS response to brain damage and degeneration. Yet the underlying neuroanatomical mechanisms remained to be established and the respective pathway effects were only conveyed through the discovery of neural stem cells in in adult mammalian brains in the early 1990s. Experimental results have since emphasized the genuine existence of adult neurogenesis phenomena in the CNS. The focus in this essay will be laid here on questions of the structure and function of scientific concepts, the development of research schools among biomedical investigators, as well as the impact of new data and phenomena through innovative methodologies and laboratory instruments in the neuroscientific endeavors of the 20th century.

## Introduction

From a medical history and history of science perspective alike, the development of the research concept of “brain regeneration” (or “brain plasticity”) is of great and persisting interest. It allows us to study questions of scientific methodology, social dimensions of neuromorphological investigations, as well as the medical history connections with recent problems in the clinical neurosciences and in the context of bench-side regeneration research (see also Stahnisch, 2003). For the purposes of this review essay, of course, only several limited (albeit instructive) historical vignettes can be provided since the problem area is so diffuse that many monographic scholarly books have already been published on the topic ranging from historical (Doidge, 2015) and sociological (Jacobson, 1993) over to anthropological perspectives (Rees, 2016). Out of the more than ten thousand journal articles and hundreds of textbooks published on the topic of brain (CNS) regeneration phenomena, a focus had to be laid here on the century of research endeavors, beginning with Santiago Ramón y Cajal’s (1852–1934) pioneering work on neural de- and regeneration (Cajal, 1894; 1907) and ending with the discovery of stem cells in the CNS at the start of the American “Decade of the Brain” in the 1990s (Jones and Mendell, 1999). To tackle the important problem of de- and regeneration in the modern neurosciences, this historically and philosophically oriented essay is organized in three parts. First, I intend to sketch the development of the modern notion of brain regeneration and structural plasticity in broad strokes from the experimental biological approaches of the 19th century, particularly those analyzing neuromorphological concepts of interpreting degenerative and injury phenomena through clinically relevant perspectives. This includes, for example, the Frankfurt experimental physiologist Albrecht Bethe (1872–1954) (Bethe, 1903), who clinically observed and experimented with survivors of industry accidents in the 1920s and early 1930s. In several of these patients, such as in a young man who had lost his left arm while handling a production machine, Bethe realized how difficult it was to reach complete functionality through the nerves innervating the remaining *Musculus biceps brachii* and *Musculus triceps brachii*. Often, the tendency showed regenerative innervation of the antagonist instead of the agonist muscle (Bethe and Fischer, 1931).

**FIGURE 1:**
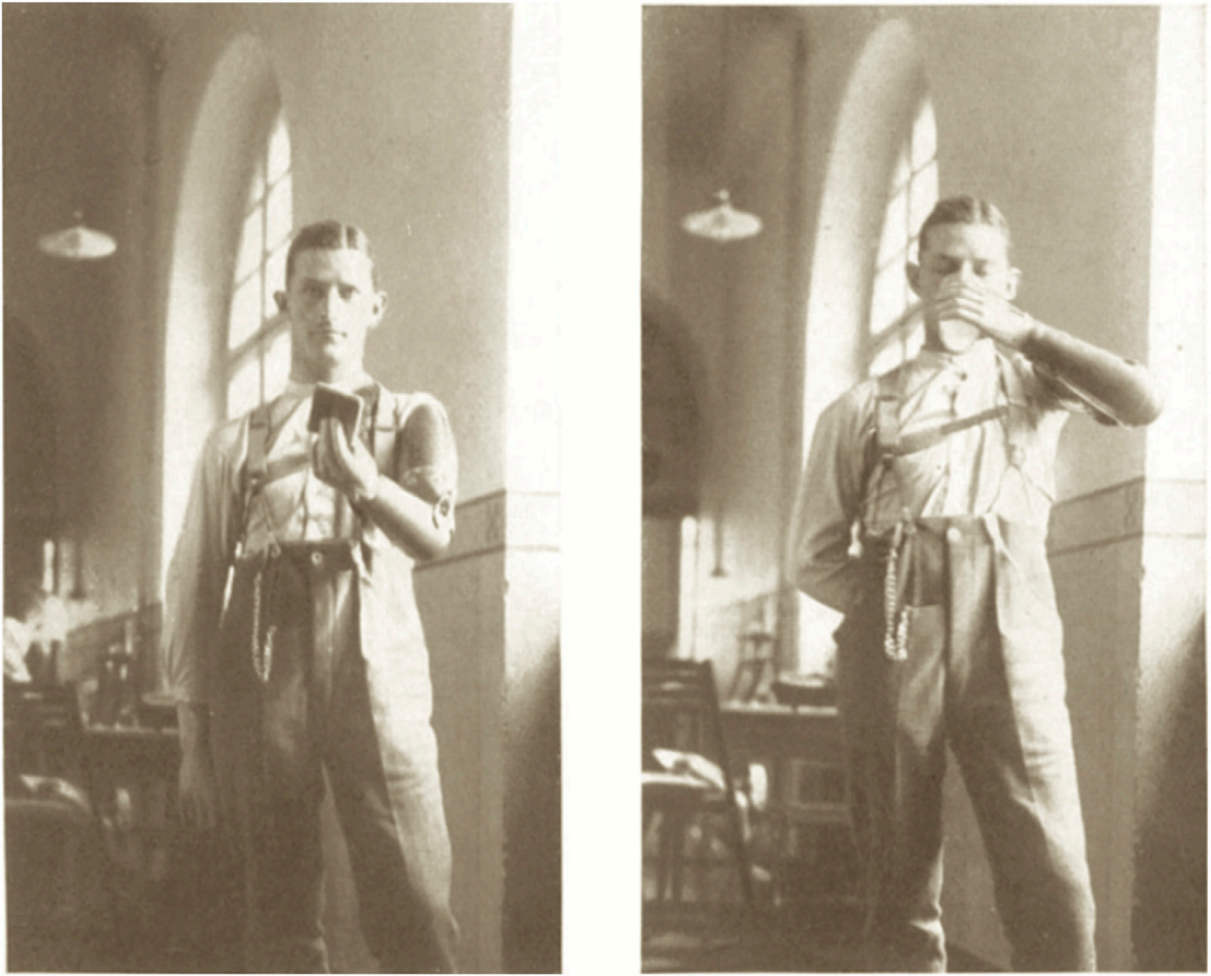
*A. Bethe and E. Fischer* (1931), *Die Anpassungsfaehigkeit (Plastizitaet) des Nervensystems. Einfuehrung und experimentelles Material,* 1112. Sketch ^©^ Public Domain.

However, when specifically asking and training the patients to think voluntarily about moving the stump of their arm, Bethe reported that with such psychosomatic interaction full functionality could be achieved over month-long rehabilitative training. This included the handling of artificial arm prostheses fixed to the remaining morphology of the arms—something that he interpreted as neuroplastic processes and functional healing (Stahnisch, 2016) (Figure 1).

Second, on the level of the functional implications for the CNS, I want to apply here an analytical framework developed by Polish-Israeli historian and philosopher of science Ludwik Fleck (1896–1961) in his *Genesis and Development of a Scientific Fact* (originally published as Fleck, 1935). This will allow for including some of the social conditions which experimental and clinical researchers faced, which influenced the divergent interpretations of de- and regeneration phenomena in the human brain, ranging from the traditional dogmas of the structural rigidity of the CNS to the recognition of neuroplasticity and neurogenesis. This review essay further seeks to examine the practical implications of theory dynamics regarding new and contingent model organisms of regeneration phenomena that furthered morphological research advances along innovative scientific trajectories in modern biomedicine. Fleck’s concepts and theoretical insights can help to investigate traditionalist views among groups of brain researchers and neuroscientists in addressing new phenomena emerging in a social context of uncertainty. Resulting group-based “thought styles” strongly influenced and shaped the acceptance of new ideas regarding “structural” and “regenerative” plasticity in the adult human brain.

Third, I will argue that the history of “brain regeneration phenomena” over the period analyzed here displays two important assumptions by Fleck about the nature of “thought communities” (brain scientists who advocated for or against the existence of structural neuroplasticity) as a superseding processes, yet also in a concerning manner tied to the “harmony of illusions” (the internal agreement with one preferred working hypothesis—here in neuroscientific thought communities) (Fleck, 1979, p. 38f.). Such harmonies of illusions existed between and across specific disciplinary-bound thought styles about the nature, extent, and applicability of brain regeneration phenomena for many decades, involving scientific communities from endocrinology and stretching over to neuroanatomy (Breidbach, 1997, pp. 96-99). Analyzing the historical vignettes in this review essay and teasing out the epistemological and communicative stumbling blocks and challenges can nevertheless help alleviating some of the existing difficulties in neuroscientific research trajectories regarding brain regeneration phenomena. It may also emphasize the need to develop new and much needed investigative styles of neurophysiological research.

The thesis of regeneration in the brain (or: “neural plasticity” and “structural plasticity”)—to which I will be referring to here synonymously to reduce the complexity of the topic at hand–and its relation to injury, tissue damage, and degenerative cell death can hardly be belittled regarding its scope in modern clinical and basic neurology (Dinsmore, 1991, pp. 101-112). It stretches conceptually from neuromorphology to areas of physiology and psychology, as Harvard-based clinical neuroscientist Peter R. Huttenlocher (1931–2013) described in his widely received textbook on *Neural Plasticity* (Huttenlocher, 2002):

“Neural plasticity–the brain’s ability to change in response to normal developmental processes, experience, and injury—is a critically important phenomenon for both neuroscience and psychology. Increasing evidence about the extent of plasticity—long past the supposedly critical first 3 years–has recently emerged.” (Huttenlocher, 2002, p. 194).

## Developmental Biological Approaches since the 19th Century

The subject area of brain regeneration phenomena is a highly complex one, including processes of myelin sheath reconstruction, axonal sprouting, nerve cell apoptosis, and synaptic regeneration of neuron connections (Nagappan et al., 2020). Strictly speaking, we aspire to reach at a knowledge of regeneration which can be charted, so that insights into the historical uses, conceptualizations, and awareness of the diverse processes of de- and regeneration in their respective times (MacCord and Maienschein, 2021, p. 2), rather than focusing only on a post-1990s reframing and new understanding of stem cell and genetic interpretations of neuroregeneration alone (Wang et al., 2018). Furthermore, the complexity in related physiological processes along with the wide variety of the necessary experimental research methodologies lies at the exploratory center of this historical essay (Maienschein, 2009). Out of the large complexity of phenomena linked to the notion of brain or neural plasticity I will primarily concentrate on the neuroanatomical (or neuromorphological) tradition regarding the central nervous system in mammalian and non-mammalian vertebrates (Chapouton et al., 2007). The development of the modern concept of “neural regeneration,” when widely conceived, can be traced back to the early integration of brain science and neuroanatomical research traditions since the mid-nineteenth century (Gilbert, 1992, pp. 117-145), for which the emergence of the comparative anatomical school of Carl Gegenbauer (1826–1903) in Heidelberg can be seen as a good example, since it integrated comparative with embryological approaches. This particularly regarded Gegenbauer’s emphasis on the importance of embryological developments for both phylogenetic reconstructions and restorative neuromorphological processes in the injured brain (Laubichler, 2003). Yet the research trajectories remained often separate from one another since observations about regenerative processes (such as the swelling of nerve buds, axonal sprouting, and myelin sheath repair) became likened and compared to different stages in the embryological development of the brain. These included particular cellular and subcellular details of neural migration, cell elongation, and dendritic arborization, rather than leading to investigations of “brain regeneration” or “structural plasticity” directly. It is therefore interesting to see how in a wider biological context of experimental regeneration research, such as Wilhelm Roux’s (1888) research program that physiologically investigated the regenerative abilities of individual parts of the body that were integral to the functioning of the whole organism in response to injury, Roux noted:

“Regeneration is the re-establishment of amputated limbs and other thoroughly developed parts of the body that have been lost, i. e. it is a restitution process. […] Regeneration is brought about mechanically, after Roux [he referred to himself in third person singular], because the cells of the fully developed body entail somatic germ plasma […]. And the particular kind of defect brings about the necessary supplementation from this omnipotent [biological] stock.” (Roux, 1888, p. 18f.).

The early experimental paradigms of the time that were primarily based on surgical methods of amputating individual body parts and ligating principal and thus controlling morphological structures had already led to knowledge about the biological dispositions for regeneration found in the so-called “lower animals,” such as worms, sea urchins, crustaceae, mollusks, or cephalopods (Nakajima et al., 2018). These approaches included experimental observations such as the incomplete foot regeneration in *Hydra* or the full regeneration of pincers in river crabs as the Baltic German zoologist Nicolaus Kleinenberg (1842–1897) (Kleinenberg, 1872) and the German embryologist Curt Alfred Herbst (1866–1946) (Herbst, 1900) had observed. Moreover, a series of monographs appearing at the beginning of the 20th century drew attention to the burgeoning research area of biological de- and regeneration, primarily in the PNS, but also regarding degenerative pathological processes in the CNS—including the contributions by the American evolutionary biologist, and Nobel prize laureate of 1933, Thomas Hunt Morgan (1866–1945) (Morgan, 1901), German zoologist Eugen Korschelt (1858–1946) (Korschelt, 1907), and Austrian biologist Hans Leo Przibram (1874–1944) (Przibram, 1909).

Yet when their experimental laboratory approaches are examined, they were consistently rather heuristic, schematic, and not very precise, since the contemporary paradigms included such crude approaches as the decapitation of full animal heads or viewing the eyes of test animals as protracted and thus easily accessible brain parts that offered points of surgical entry for extirpation and ablation experiments. Thomas Hunt Morgan, for example, witnessed (in 1901) that *Planaria*, which had been experimentally decapitated directly behind their eyes, would regenerate a second head and yet did not anatomically rebuild the postencephalic regions (Jahn, 2000, pp. 444-485).

**FIGURE 2:**
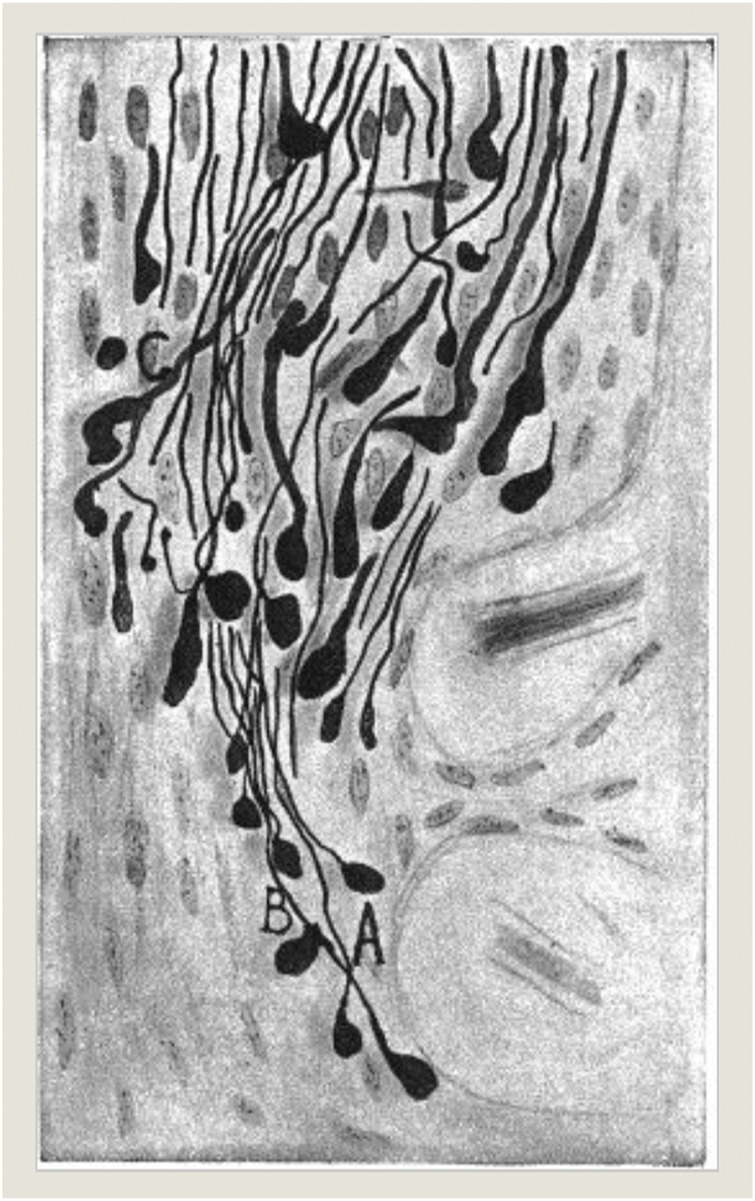
*J. F. Tello* (1911): *“La influencia del neurotropismo en la regeneración de los centros nerviosos,”* 125. Photograph ^©^ Public Domain.

Working experimentally at the intersection of the optic chiasm like Hunt Morgan’s experimental models, Spanish neurohistologist Jorge Francisco Tello Muñoz (1883–1959) realized 10 years later that nervous sprouting did happen in optic nerves which had been surgically cut in pigeons as research models (Figure 2). He concluded that such occurrences 3 days following the experimental severance needed to be interpreted as primarily degenerative in nature when such drastic artificial injuries occurred (“*La influencia del neurotropismo en la regeneración de los centros nerviosos*;” Tello, 1911). Similarly, in his own interpretations of the significance of nervous sprouting, Tello’s mentor at the Laboratorio de Investigaciones Biológicas in Madrid, the later Nobel prize winner and founding figure of neuroscience Cajal remarked about the brain’s regeneration properties:

“Pathologists consider it an unimpeachable dogma that there is no restoration of the central paths [the CNS], and therefore that there is no restoration of the normal physiology of the interrupted conductors [the nerve fibers] in the spinal cord. A vast series of anatomico-pathological experiments in animals, and an enormous number of clinical cases that have been methodologically followed by autopsy, serve as a foundation for this doctrine, which is universally accepted to-day.” (Cajal, 1991, p. 509).

**FIGURE 3:**
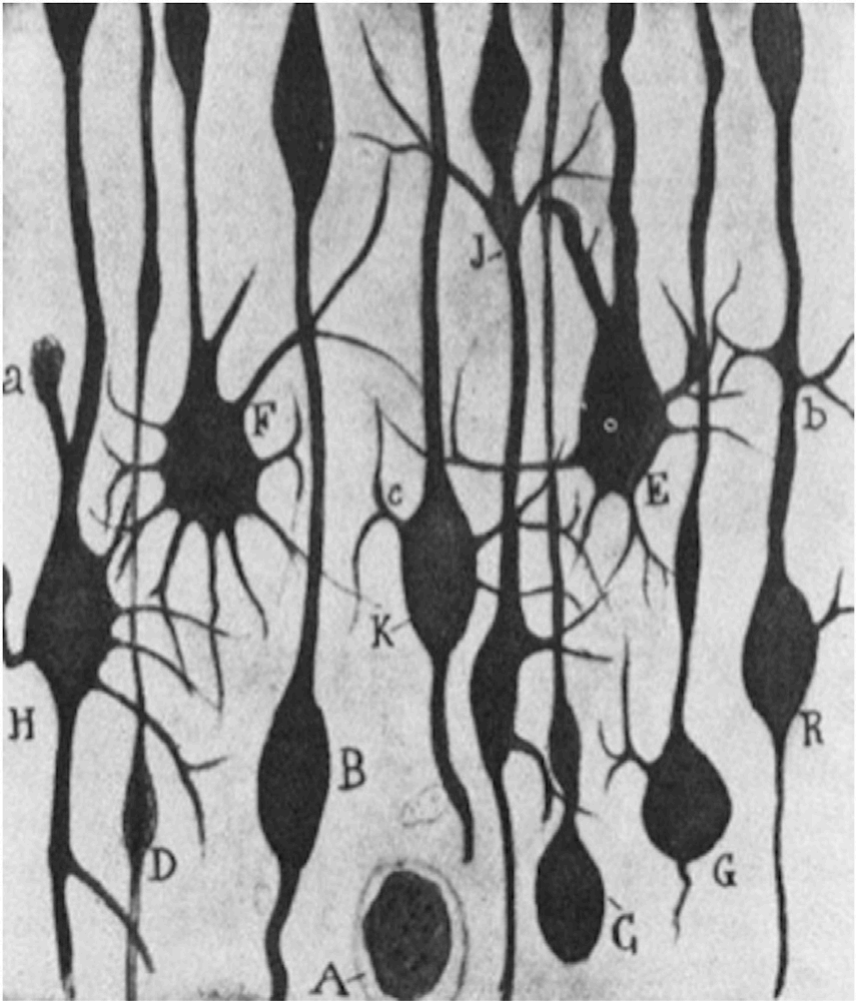
*S. R y Cajal* (1928): *“Estudios sobre la degeneración y regeneración del sistema nervioso,”* 57. Ink drawing ^©^ Public Domain.

This perspective put forward by Cajal was however not solely a judgement grounded in a review of the existing literature at the time but was likewise based on active laboratory research pursued on regeneration as is seen in his book *Regeneración de los nervios* (Cajal, 1907). There, he identified “aberrant sprouts” in the motor cortex of a two-day-old dog which had been experimentally ablated in the frontoparietal cortex, following the test animal’s being euthanized and pathologically dissected after 24 h. Also, comparative lesions in the motor cortex of a cat could give rise to “hypertrophic arctiform collaterals” which Cajal likened to aberrant growth phenomena as a result of the foregoing artificial cortical destruction (Figure 3).

Cajal’s last surviving pupil Dr. Carlo Léoz Ortín (1879–1990) had frequently expressed that even Cajal could be enormously paternalistic and dogmatic about his views on neuronal regeneration (Bergua-Aznar, 1988)—despite his meandering course taken as to what the biological regeneration phenomena could generally mean (Finger and Stein, 1982, pp. ix-xi). In following Cajal, a multitude of prominent neuropathologists and neurologists endorsed the traditional dogma according to which the CNS displayed an unchanging neural set-up which proved to be incapable of building new nerve cells for the restoration from brain or nerve damage. Yet some contemporary neurologists and morphologists continued to endorse the view that axonal growth properties existed that gave rise to local sprouting mechanisms that could compensate for some of the neurological injuries experienced (e.g., Nageotte, 1906; Marinesco, 1910; Spatz, 1930).

*As an intermediary resumé*, we could state at this point that the tenants of biological regeneration research at the end of the 19th to the beginning years of the 20th century were characterized by the assumption that inherited dispositions for axonal growth and structural nerve repair existed in the research organisms that they used as experimental starting points for their laboratory investigations (Stahnisch, 2019). However, the environment was already seen as a landscape full of influencing biological and social factors which could be further understood through contemporary—though obviously rather makeshift and experimental—procedures that involved surgical extirpations and ablations, ligatures, and transplantations—all being pursued with the aim for better understanding and mastering biological regeneration phenomena with future medical applications for human patients in mind (cf. Pauly, 1987).

Regarding analytic perspectives on the human brain at the turn of the century, Cajal’s studies of the intimate neural structure of the hippocampal formation from the 1890s onward can be seen as standing out from those of other contemporary neuroanatomists or neuropathologists (e.g., Cajal, 1892). In his experimental series, he in fact concluded that the cortical anatomical organization of the human hippocampal formation could be understood in terms of an embryological developmental “involution” of this part of the temporal lobe which would relate and liken neural regenerative processes to those of neural growth and from there to those of normal neuromorphological formation (Shepherd, 1991, pp. 243-271). His own experimental investigations with the help of refined Golgi stains and his own adjustments and advancements of them into the precise de- and regenerative phenomena displayed in the hippocampus in cats, dogs, and in mice only appeared later during the interwar period of the early 20th century (Hagner, 1999, p. 180f.).

Similar experimental systems were also applied outside of the context of the leading brain science center of the Laboratorio de Investigaciones Biológicas in Spain (Mateos-Aparicio and Rodríguez-Moreno, 2019), for example in the neurohistological research of Max Bielschowsky (1869–1940) in Germany. In what was first known as the Biological Station of Oskar Vogt (1870–1959) and Cécile Vogt-Mugnier (1865–1962) and later became the world’s largest brain research center in the 1930—as the Kaiser Wilhelm Institute for Brain Research in Berlin (Stahnisch, 2020, pp. 16–21)—neuropathologist Bielschowsky was able to further identify axonal sprouting processes in human brain gliomata’s (as cancerous growths composed of cells originating from neuroglial tissue) marginal zones when applying the reduced silver staining technique that he had developed at the beginning of the 20th century. Much like Cajal, however, he initially took an ambivalent stance in thinking that the phenomena he had observed were likely “functionally meaningless” (Bielschowsky, 1909, p. 149).

## Neurohistological Staining Approaches around the Mid-20th Century

In the wake of such pioneering neurohistological studies of brain regeneration of the first half of the 20th century, quite a flurry of new staining and microscopical methods as well as experimental embryological investigations emerged to help with the study of neural sprouting and plastic processes. On these, émigré German-American developmental biologist Viktor Hamburger (1900–2001) later remarked:

“We were in awe regarding the elegance and the high skill level in the experimental practice of this master of the art [German embryologist Hans Spemann, 1869–1941]. Yet at the same time, we had not been conscious of the existing imbalance between the enormous complexity of developmental processes and the constraints of the few technical approaches, which were available at that time, including the extirpation, the transplantation, and the explantation (as an *in vitro-culture*).“ Viktor Hamburger (1990), vii [transl. & emphasis F.W.S.].

**FIGURE 4:**
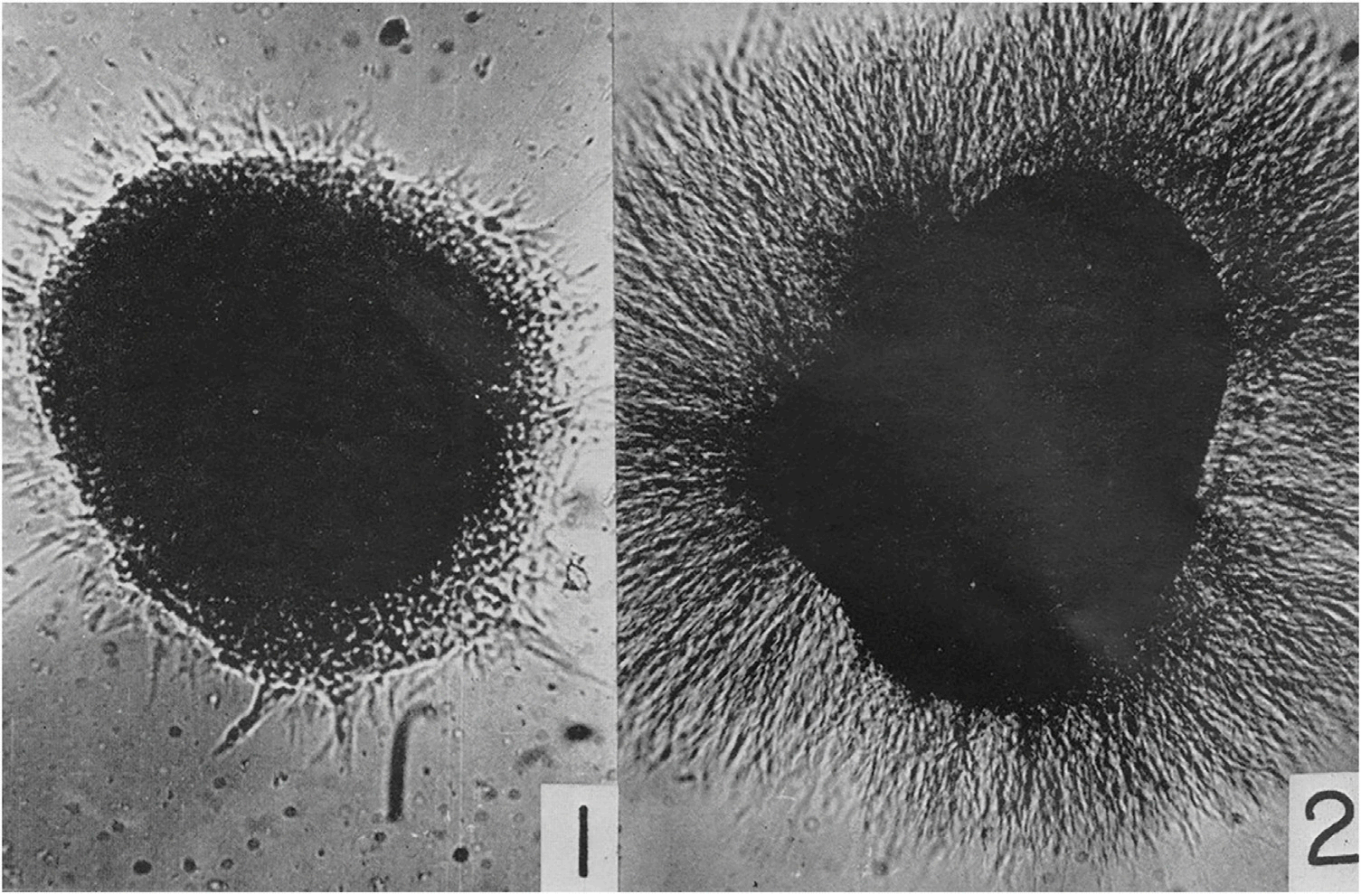
Cohen, S. and levi-Montalcini, R. (1956). “A nerve growth-stimulating factor isolated from snake venom,” 572. © Public Domain.

Due to the limitations of the scope of this article, I can only mention a few of the new “technical approaches” used to discern the “enormous complexity of developmental processes,” including Rita Levi-Montalcini’s (1909–2012) work on the physiology of nerve growth factors beginning in the 1950s (Cohen and Levi-Montalcini, 1956). She collaborated with Viktor Hamburger studying the phenomenon of venomous growth at Washington University in St. Louis and with Stanley Cohen (1942–2013) at Vanderbilt University, finding that nerve tissue from chicken embryos cultured with snake venom led to a dense *halo* of nerve axons outgrowth. Without this nerve growth factor, not as many nerve axons developed and those that did indeed grow were much smaller in size (Figure 4).

During the following decade, a breakthrough also emerged from functional neurophysiological work with the discovery of long-term potentiation (LTP). Terje Lømo (b. 1935) had worked for his doctorate in Per Andersen’s (1930–2020) laboratory in Norway where he researched the physiological results of stimulating the accessible perforant path to the hippocampus’s dentate gyrus in anaesthetized rabbits. This allowed for the serendipitous observation that repeated stimulation intervals led to growing and lasting transmission rates within perforant path-granule cell synapses (Bliss and Lømo, 1973). Together with Tim Bliss (b. 1940), they then sat out to research the long-term field potential changes as a physiological candidate for memory mechanisms in 1968, something which became the basis for the plasticity mechanism of LTP (Craver, 2007). This also included the realization of pathway specificity, electrophysiological saturation, as well as a rise in the coupling of the synaptic potentials in relation to the histological level of different discharging nerve cells, such as the population of the granule cells (Lømo, 2003).

These physiological findings—though different in biological kind—were nevertheless partly grounded in the earlier assumption of Canadian neuropsychologist Donald Hebb (1904–1985) in Montreal who had hypothesized that structural plasticity of the Cajal kind could likewise be seen as a general anatomical mechanism and substrate for human psychological learning processes (cf. Bradley et al., 1985, p. 21; Benton, 2000). This hypothesis was further validated by the ground-breaking electronmicroscopical laboratory work that played a substantial role in furthering a modern understanding of minute neural structures, such as synapses, of the British scientist Sanford L. Palay (1918–2002) (Palay, 1958) and British anatomist George Gray (1924–1999) (Gray, 1959). Electronmicroscopic research regarding nervous regeneration had previously encountered several obstacles, such as the low optic resolutions that often could not fully identify synaptic contacts or relate them to individual neural cell types (Rasmussen, 1997, pp. 164–170). Yet neurohistologists Palay and Gray were able to structurally establish the existence of axonal outgrowth phenomena together with the functionality of newly built synapses as processes of nervous regeneration in rat and mouse cortices. They further joined an illustrious international group of researchers invited by biophysicist Francis O. Schmitt (1903–1995) at the Massachusetts Institute of Technology in the United States. Out of this Neuroscience Research Program evolved an innovative network and research platform of laboratory and theoretical scientists, as well as clinicians, who were eager to arrive at interdisciplinary insights regarding chemical, physical, and morphological investigations of the brain by also including functional knowledge from new behavioral, psychological, and neuropsychiatric outcomes (Maxson-Jones, 2020). Their findings were subsequently corroborated through the well-known physiological memory and learning experiments with the sea slug and gastropod mollusk *Aplysia* in Eric Kandel’s (b. 1929) laboratory at Columbia University’s medical school in New York City. These experiments proposed an intricate connection between behavioral adaptations and changes with biological substrates of memory, such as linking the experimental electrical stimulation of single motor neurons to the habituation and dishabituation effects in the functioning of gill and siphon withdrawal reflexes (cf. Kandel and Spencer, 1968).

However, new progress in bringing such structural and functional advances in neural plasticity and regeneration research ever closer together came to be rather stalled for one and a half decade due to the lack of new biological staining techniques (MacCord and Maienschein, 2021) which could have allowed for the visualization and identification of the full arborization of de- and regenerating neurons. It took in fact until the 1970s, when with horseradish peroxidase (HRP) neuroanatomical tracing techniques made new advances possible, such as the identification of fresh ascending axons that traversed and entered the distal stumps in spinal cord injuries (Suzuki et al., 2002, p. 121), of regenerating facial motor neurons, and of rebuilt retinal ganglion cell layers following ischemic damage (Young, 2009, p. 12722). The availability and wider use of HRP exerted a great influence on neuroanatomy when tracing specific pathways in the context of neuroregenerative research (Kristensson and Olsson, 1971). During the mid-1980s, further methodological changes arrived, and the new immunohistochemical staining for glial fibrillary acidic protein (GFAP), as well as radioimmunoassays such as in Pasco Rakić’s work (Rakić, 1985), became further available for neuromorphological advances in brain regeneration investigations.

**FIGURE 5:**
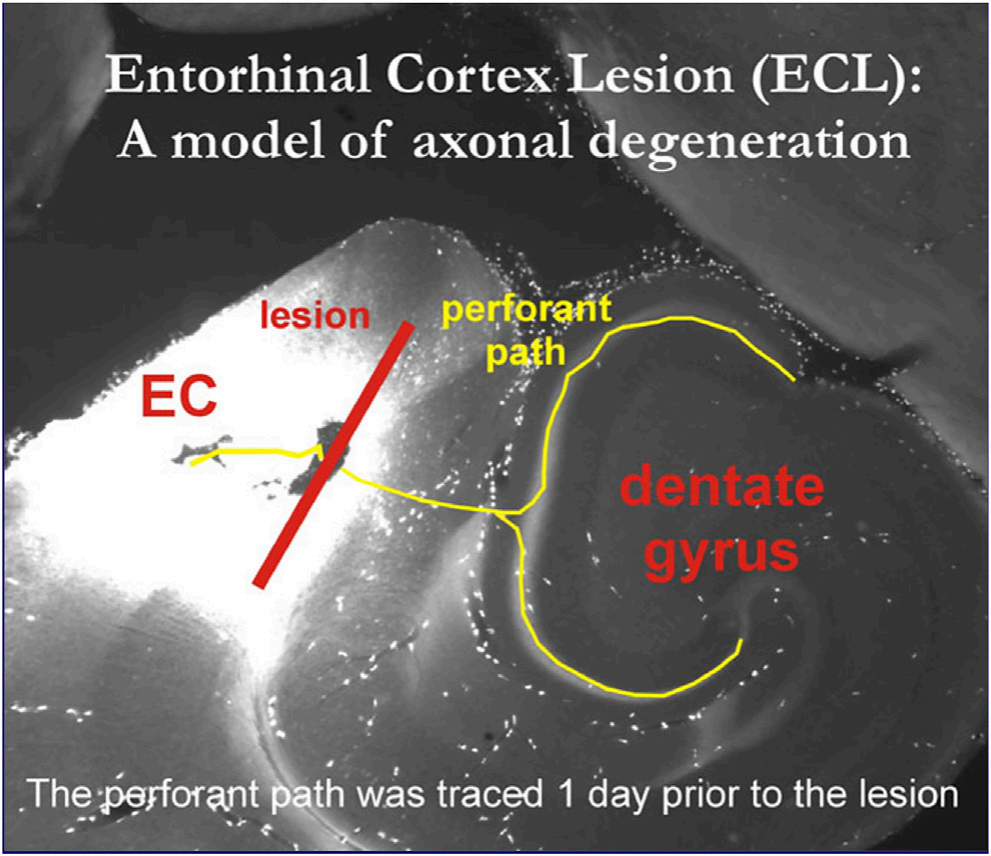
Osmicated HCN1 immunoreactive sections for ultrastructural studies of entorhinal cortex lesion model. © Personal image provided by prof. Robert Nitsch, Inst. for Anatomy, charité Berlin, Germany, 2005.

Moreover, the microscopic detection of the contribution of individual neural cell types to the establishment of synaptic contacts had to await innovative optical technologies which could be integrated with derivatives of the Italian neuroanatomist Camillo Golgi’s (1843–1926) silver staining method (Nitsch, 1988, p. 17), such as in later entorhinal cortex lesion models of axonal de- and regeneration processes in rats and mice that visualized respective structural processes alternatingly with Golgi staining and immunofluorescence methods (Deller et al., 1996), e.g., osmicated sections for ultrastructural studies with electron microscopic images that could show the fine details of sprouting and synaptic contact formation (Figure 5).

## The Social Conditions of Experimental and Clinical Researchers

In addition to the technological advances as well as practical transformations in the research methodologies for brain regeneration, the Figure 5 transformations in the social conditions and the emergence of different cultural contexts of neuromorphological research advances into brain regeneration phenomena need to be considered and historically examined. They have established important research grounds for early regenerative concepts and programs in experimental neuroanatomy, neuropathology, and clinical neurology at the beginning of the 20th century. Such brain research developments can certainly not be regarded as isolated from broader societal developments. This can also be gleaned from the discourses on social de- and regeneration, neurasthenia, nerve-weakness, and the experiences of the brain-injured before and after WWI (Stahnisch, 2009a). Moreover, when adopting an earlier epistemological position by Ludwik Fleck for the historiographical analysis of the development of scientific facts and biomedical knowledge, Austrian sociologist Karin Knorr-Cetina has dubbed such peculiar amalgamations between the scientific and cultural spheres a “condensation of society in the experiment” (Knorr-Cetina, 1988, p. 85).

The period between the 1890s aand 1990s is particularly suitable for such a historical research endeavor because earlier discourses on the “mental and physical degeneration” of modern man (cf. Morel, 1857/1858) became ever more prominent under the new societal conditions at the end of the 19th century, during WWI, the interwar period, as well as throughout and after WWII. “Degenerative views” in neurological and psychiatric theory respectively underpinned widespread cultural beliefs about what German historian Joachim Radkau termed *The Age of Nervousness* when researching and analyzing German history between the political ascent of Otto von Bismarck (1815–1898) and the end of the National Socialist Period (Radkau, 1998, pp. 9–15). Awareness of such cultural tropes can provide a cultural appreciation for the practical and social working contexts of laboratory brain scientists as their physician and anthropologist peers began to move the wider cultural meanings of de- and regeneration into a semantic field of rehabilitation medicine (Zeiter, 1954).

The pediatric neurologist Michael E. Selzer from Philadelphia has examined the semantic domain of morphological degeneration and regeneration phenomena in the nervous system in its historical development towards the medical subspecialty of neurorehabilitation (Selzer et al., 2014, pp. xix–xx). Similar to Radkau’s analysis of the Central European medical research context (Radkau, 1998, pp. 81–129), Selzer has drawn scholarly attention to the significant group of brain-injured veterans from WWI and WWII, which necessitated the creation of a new community of physical therapists for war veterans’ retraining and resocialization into the existing, productive workforce in North America. It crystalized in the societal fusion of the early American College of Radiology and Physiotherapy (founded in 1923) and the American Congress of Physical Therapy (inaugurated in 1925) in 1945, when the American Congress of Physical Medicine and Rehabilitation was created at the end of WWII (Zeiter, 1954, pp. 683–688). This new medical specialty underscored the need for physical treatment approaches, which comprised occupational and physical therapy, electrostimulation, diathermy, massage therapy, as well as thermo- and cryotherapy (Selzer et al., 2014, p. xx). It also afforded a specific role for neurorehabilitation to address the social and psychological adjustments to physical and mental forms of degeneration and disability, which involved further treatment responses to autonomic instability, decubitus ulcers, pain syndromes, urinary tract infections, along with medical problems that chronically ill patients constantly faced. The emergence of the new medical subspecialty of neurorehabilitation progressively offered answers to broader contemporary health and social problems too (Weiss, 1990).

For the historical argument of this essay, the above overview serves to illustrate a free-floating culture of ideas, practical experiences, and organizational skills which pertained in the field of neuroregeneration research from its beginnings to the modern situation regarding neural repair and rehabilitation:

“Neurorehabilitation services are complex multifaceted, multiprofessional systems. Without systematic structuring of the treatment processes involved, a high degree of variance in provision of the service across staff members, patients, and time is likely. […] The continuous critical evaluation and updating of clinical pathways will improve the provided care further. It is suggested that clinical pathways for neurorehabilitation services should not be prescriptive but should respect the need for comprehensive assessment of individual needs and a customized rehabilitation program designed under the supervision of a consultant, while, at the same time, provide standards for documentation, communication, and therapeutic interventions.” (Selzer et al., 2014, pp. 57–76)

As described by Selzer, in these “multifaceted and multiprofessional systems,” the extraordinary increase of staining and microscopic methodologies for the purposes of neuromorphological neuroregeneration research is included. Such evaluation and adjustment also extended to the investigation of *in vitro* tissue cultures when de- and regenerative specimens and histological nerve lesion preparations were prepared in particular time courses (see Harrison, 1907). This anatomico-mechanistic tradition, over one century, also shifted more and more to specific clinical problems, such as the structural and functional impact of specific neurorehabilitation processes or therapeutic neuropharmacological approaches and treatment review assessment (Morgan, 2017).

From the late 1950s, neuromorphologists examined, with even greater interest, the physiological course of degeneration and regeneration phenomena following major brain damage by applying their revised and augmented microscopic staining tools. Dutch-American anatomist Walle Nauta (1916–1994) at the Massachusetts Institute of Technology and the Swiss chemist Paul A. Gygax (d. 1969?) at the University of Zurich (Nauta and Gygax, 1954) created a significantly innovative histological silver stain after having observed in substances which had been imbued with silver that it could render visible specific anatomical alterations ensued from harmed nerve axons and arborizations (Switzer, 1991, pp. 91–92). One may also realize, regarding their contemporary research context, that societal concerns regarding nervous degeneration, cultural metaphors of “exhaustion” and “consumption,” as well as shifts in the research context from war injuries to socio-rehabilitation research had become profoundly inscribed in the socio-technical experiments of neurohistological regeneration research (Stahnisch, 2009b, pp. 29–32).

This extraordinary research trend can also be found in the neuroscientific programs supported by the intramural and extramural funding programs of the National Institutes of Health, which took over the important and globally leading funding role for biomedical research from the Rockefeller Foundation after the WWII (Hollingsworth, 2004). The scientific administrators at the National Institute for Neurology and Blindness and at the National Institute of Neurological Disorders and Stroke specifically hoped for clinically relevant translational research successes–especially for the benefit of patients with spinal cord injuries (Farreras et al., 2004, pp. 19–32) aligned with funding from the Paralyzed Veterans of America organization since 1946 (Fonseca et al., 1996). This development became visible in the fact that since the 1950s, the National Cancer Institute and the National Institute of Child Health, for instance, supported the aforementioned experimental development-oriented research by Rita Levi-Montalcini regarding the mechanisms of nerve growth factor(s) in which she had researched venomous growth and was able to establish that nerve tissue surgically excised from chicken embryos and exposed to snake venom led to processes of nerve axons (Cohen and Levi-Montalcini, 1956).

Additional important research funded by the National Institutes of Health were for example the immunoflourescence marking and staining techniques, including those in the fundamental work by cell biologist Elizabeth H. LeDuc at the National Institute of General Medical Sciences from the 1950s to the 1970s (LeDuc and Bernhard, 1961). Later, these became widely included in combined light and electron microscopic study models of axonal de- and regeneration, such as was seen in the perforant path entorhinal cortex lesion model used by neuroanatomist Robert Nitsch’s research group at the Charité Medical School in Berlin during the 1990s (Nitsch and Frotscher, 1992).

From the historical course of research activities outlined and described in this essay so far, one could arrive at the expectation that experimental neuromorphological research advances regarding brain regeneration phenomena would have progressed steadily, in an orderly fashion, and solved the challenging questions in anatomical and cell biological laboratories one by one, without running into impasses, experiencing scientific road blocks, or being subject to any scholarly debates about the course of action. Yet while the nature of research programs in regeneration research and medicine is well known to Science, Technology and Society as well as History and Philosophy of Science scholarship (Jacobson, 1993; Craver, 2005), two main research questions emerge from here:

First, the social and cultural environments of medical regeneration research are fairly unaddressed and figure as a desideratum in interdisciplinary scholarship.

The above-mentioned (Table 1), extended social contexts are often referenced in the related scholarly literature but not fully explored regarding their impact on neuroregeneration research.

**TABLE 1:** The social environments of medical regeneration research still appear as a desideratum in interdisciplinary scholarship (Table 1).

Degeneration discourse	→	General biological nature of neuroregeneration
Rehabilitation discourse	→	Concern with social/environmental factors
Post-WWI/Post-WWII wounds	→	Peripheral and central regeneration of war
Creation of the National Institutes of Health	→	Cancer, demographic transition, veterans’ paraplegia
Interdisciplinary organization	→	Military and industry interests in neuroscience

Moreover, following from these exemplary “external” contexts of regeneration research, a second main research question emerges about the epistemological exchange relationship and reciprocity of medical theory dynamics. The genesis of new social and ethical questions needs to be much further addressed too, as will be pursued in a methodologically illustrative way in the following part of this essay.

## “Brain Regeneration Phenomena” as Seen Through Several of Ludwik Fleck’s Assumptions About the Nature of “Thought Collectives”

Ludwik Fleck had intriguingly analyzed intellectual and organizational influences in his book *Genesis and Development of a Scientific Fact* (translated into English by American sociologist of science Frederick Bradley and the Polish-American medical philosopher Thaddeus J. Trenn, 1937-2013, in 1979). Most legendary among them are Fleck’s previous analyses of epistemological questions, theory changes, and “*Gestalt* switches” (an immediate perception change, including changes in scientific perspective) from what he called research-based pre-ideas to novel scientific “facts” in oncology, immunology, hematology, biomedical diagnostics, and so forth (Loewy, 1990, pp. 215–228).

Based on such traditional lines of history and philosophy of science analyses, I want to examine the first phase of neurode- and neuroregeneration research between the 1890s and early 1960s through the perspective developed by Fleck’s well-known model of a succession of “thought collectives” (brain scientists who advocated for or against the existence of structural neuroplasticity) employing his definition:

“A truly isolated investigator is impossible […]. An isolated investigator without bias and tradition, without forces of mental society acting upon him, and without the effect of the evolution of that society, would be blind and thoughtless. Thinking is a collective activity […]. Its product is a certain picture, which is visible only to anybody who takes part in this social activity, or a thought which is also clear to the members of the collective only. What we do think and how we do see depends on the thought-collective to which we belong” (Fleck, 1979).”

The research period from the 1890s to the early 1960s in brain regeneration investigations can historically be regarded as one characterized by the succession of the “brain rigidity dogma” through to the acceptance of a “plastic regeneration thought style” (cf. Kandel et al., 1991). During Cajal’s time at the turn of the century–when several indications of regenerative processes existed in related brain science scholarship, and when even Cajal had been vacillating considerably on this point in different publications regarding the restorative or aberrant processes of degeneration and regeneration in central nervous system neurons (Stahnisch and Nitsch, 2002)—the research direction which sought to reduce physiological assumptions to morphological entities had faced serious epistemological problems. For example, it remained very difficult to establish a satisfactory correlation between varying cell types in the brain, such as neurons, oligodendrocytes, and astrocytes, as well as the present individual functional systems, at a time when Cajal and other brain scientists failed to establish robust functional and physiological interpretations of the observable de- and regenerative structural changes in the histological architecture of the central nervous system (Cajal, 1894, p. 87):

“Some, which doubtless are centripetal and therefore in continuity with uninjured cortical neurons of other cerebral lobules or of the optic thalamus (ascending sensory fibers). But since all are undergoing traumatic degeneration, it is impossible to differentiate physiological or topographical categories of conductor.” (Cajal, 1991, pp. 649–650).

Cajal’s interpretation, however, represented new lines in the thought style of the brain sciences that ensued in the scientific community, *viz*. the theoretical merger of several existing approaches that had their scientific roots in clinical neurology, behavioral psychology, and developmental psychology (e.g., Gage et al., 1982; Cotman and Nieto-Sampedro, 1985). A review of this very active period in the history of neuroregenerative research in the central nervous system is so intriguing and stimulating precisely because the assumption that structurally and functionally adequate processes of regeneration genuinely existed in the brain had had “no good press” for over 60 years.

Yet in what was to follow through Geoffrey Raisman’s (1939–2017) experimental investigations at University College London on the Septum (Raisman, 1969) and Carl W. Cotman’s work at the University of California at Irvine on the Hippocampus (Cotman and Nadler, 1978), the interest in brain regenerative research was about to surge in fully unknown ways. Prominent neurohistologist Raisman could for instance show in his electron microscopy research in the laboratory, using rats as his experimental models, that collateral forms of axonal sprouting occurred after incomplete surgical denervation of the septal nuclei. His rationale for experimentally lesioning the septofimbrial system was based on its neural input from two diverse tracts, namely through the hippocampal formation and hypothalamus that structurally merge in the forebrain bundle. When looking at the structural conditions in the brain, almost half of the nerve axons happen to stem from the contralateral hemisphere–something which offered taking a double-lesion approach (Raisman, 1969, p. 1973). As a result of his anatomical investigations of the septofimbrial system, Raisman reached the now more widely observed and accepted conclusion that “the anatomical structure of the brain was by no means rigid” (Raisman, 1978, p. 104). With this assessment, he was Raisman was however one of the early and more modern neuroscientists, who provided neuroanatomical proof of the occurrence of “neuroplasticity.” It was grounded in his early work using a morphological lesioning model, while investigating neural repair as a structural regeneration process that followed reconstruction of the axonal projections in the septofimbrial system.

During the early 1980s, further significant neurogenesis could be shown in the vocal regulator nucleus in the canary central nervous system (Fuchs and Fluegge, 2014, p. 6). This also allowed for forming a functional interpretation between bird behaviors, their aptitude regarding song learning, as well as the development of neurogenesis (Álvarez-Buylla et al., 1988, pp. 8722–8724). The resulting observations that songbirds, including zebra finches and canaries, showed morphologically increased vocal regulator nuclei in their central nervous system suggested that the neuron counts in the tested adult songbirds correlated with the respective times of the year “as a critical period” (Nottebohm and Arnold, 1976, pp. 211–213). These research initiatives around the groups of investigators led by Argentinian-American neuroethologist Fernando Nottebohm (b. 1940) at Rockefeller University in New York City and Mexican-American developmental neurobiologist Arturo Álvarez-Buylla (b. 1958) at the University of California at San Francisco’s Brain Tumor Center could show that the neural cell count in songbirds’ vocal regulator nuclei rose during spring, when male canaries and finches commence singing to instigate courtship and incubation behaviors. Newly generated neurons could be found in these songbirds’ hyperstriatum ventrale, pars caudalis brain region (Nottebohm, 1989, pp. 74–79). Investigations of the neurons in the hyperstriatum ventrale, pars caudalis resulted in the realization that steroid hormones, especially the gonadal hormone testosterone, significantly influenced the processes of neurogenesis and neuroplasticity as an expression of the neuroendocrine function of the brain (Arnold and Gorski, 1984, pp. 413–442), leading to an increased interest at the beginning of the 1990s in the topic of brain regeneration (Fuchs and Fluegge, 2014, p. 3).

This change in thought styles from the widely held belief since the 19th century about a fixed location of brain functions in the hard-wired morphology of the brain to the acceptance of plastic and adaptive structural processes is also well reflected in the most central textbook in the field, *viz.* neuroscientist Eric Kandel’s and American neurobiologist James H. Schwartz’s (1932–2006) *Principles of Neural Science*. In the first edition of 1981 (Kandel and Schwartz, 1981, p. 143), it proclaimed that “neurons with processes confined to the central nervous system may undergo chromatolysis after axotomy, but they then degenerate or remain in a state of severe atrophy. This is presumably because they cannot restore appropriate synaptic connections.” This assumption had, however, changed in the course of later editions, beginning with the third edition of 1991 in which the brain’s overall capacities for neuroplastic processes became subsequently highlighted as well: “Through the use of tissue slice techniques, cell and molecular biological approaches can be applied to virtually any part of the mammalian brain. Information obtained from recordings made in brain slices has provided important insights into such problems as synaptic plasticity, the mechanisms of epilepsy, and the actions of drugs on the brain” (Kandel et al., 1991, p. 788).

The new windfall in medical research funding—during the third phase of neurode—and regeneration research from the 1990s to the 2020s—provided a sound foundation for a stark increase in the research activities of brain science through the important decade from 1990 to 2000 under US President George H. W. Bush (1924–2018). Popularly known as the American “Decade of the Brain” (Albright et al., 2000), the time around the turn to the new millennium saw a renewed and remarkable rise in interests in many neuroscience activities (Lenn, 1992, p. 512f.). This extended further into American and international scientific collaborations in brain research. At the University of Calgary in Canada, for example, Dr. Brent A. Reynolds—who subsequently moved to the United States, where he now works at the University of Florida in Gainesville, Florida—and Dr. Sam Weiss effectively discovered the existence of neural stem cells in the brain of adult mammals in 1992—a feat which became synonymous with pivotal discoveries in brain science for the rest of the decade (Reynolds and Weiss, 1992). The resulting article provided a pivotal example of the important experimental and histological work being done in the neuromorphological research area regarding brain regeneration phenomena and eventually it led to the recognition of scientific excellence through the bestowment of a national Canadian Gairdner Award in 2008 (Lampard et al., 2021, p. 154). Through Weiss’ and his research group’s first discovery of neural stem cells in the adult human brain, they helped solve the major problem in the history of neuroanatomy regarding the existence and mechanism of structural plasticity in the human brain (Martino et al., 2011).

When synthesizing what further happened in the field of neuroregeneration since the 1990s, it has emerged from the existing literature that the capacity of the adult human brain to restore function from damages, such as stroke, tumors, and neurodegenerative diseases, was limited (Frey, 2001). Existing capacity for repair of neural connections, either through surviving neurons or through neurogenesis, appears not very extended in brain regions bereft of stem cells (Gould, 2007, pp. 481–483). Heterogeneity in neural stem cell occurrence and proliferation in various brain regions was also highlighted through pathological dissection material from patients, who had died from neuropsychiatric conditions such as schizophrenia, depression, and bipolar affective disorder. Reduced neurogenesis was not found in areas such as the dentate gyrus, and neural stem cell growth did not change under antidepressant drug treatment—yet significantly decreased neurogenesis could be seen in groups of schizophrenic patients (Reif et al., 2006). On the level of experimental therapy approaches (Xie et al., 2020), recent facial nerve studies need to be mentioned that have used glial cell-derived neurotrophic factor (GNDF) to stimulate the differentiation of dopaminergic neurons in facial nerve growth (Barras et al., 2009). Additional research groups have investigated the impact of the reduction of oxidative stress immediately after nerve injury, finding that it brought on increased axonal regeneration in instances of facial nerve repair (Wang et al., 2009). More studies were developed on such new understandings of neural stem cells (Zakrewski et al., 2019), attempting to provide clinical therapies for neurodegenerative diseases such as Alzheimer’s disease, Parkinson’s disease, and Huntington’s disease. Induced pluripotent stem cell-conditioned media from skin punch biopsies have for example been applied for the treatment of Alzheimer’s disease (Awe et al., 2013). While this approach is still deemed in its experimental stages, brain tissue from aborted human fetuses has been used in Huntington’s disease, albeit with mixed successes. The results have emphasized a future need for therapy approaches with pure neural stem cells (Wright and Barker, 2007). However, there still remain major hurdles for the use of stem cell applications based on the neural transplantation paradigm. Yet the stem cell-based regenerative strategies regarding the brain hold a high potential for the functional reconstruction following lesions. To further this research, clinical studies are crucial and new randomized controlled trials are needed, while the retainment of patients in such studies has proven to be socially and practically challenging (Carpenter, 2017). Multiple clinical trials, using induced pluripotent stem cells as well as human embryonic stem cells for the treatment of neurodegenerative conditions, have recently been ongoing. Yet stem cell therapies in neuroregenerative medicine remain limited through regulatory frameworks, the heterogeneity of the conditions, and the comparability of the existing studies (Henriques et al., 2019, p. 10). As a parallel observation, neurological investigations have also highlighted new and potentially neuroprotective and neurotrophic mechanisms by which neural stem cells could be beneficial for the host CNS and manipulable for future therapeutic applications (Einstein and Ben-Hur, 2008, p. 455).

When we envision the neuromorphological research progress that had been made over the preceding decades regarding brain regeneration phenomena in concluding this essay, attention should be drawn to a recent article by German neurobiologist Eberhard Fuchs at Georg August University in Goettingen and Gabriele Fluegge of the Leibniz Institute for Primate Research in Goettingen, which they published in a special issue of the journal *Neural Plasticity* on “Environmental Control of Adult Neurogenesis: From Hippocampal Homeostasis to Behavior” (Fuchs and Fluegge, 2014), emphasizing:

“Within the last 4 decades, our view of the mature vertebrate brain has changed significantly. Today it is generally accepted that the adult brain is far from being fixed. A number of factors such as stress, adrenal and gonadal hormones, neurotransmitters, growth factors, certain drugs, environmental stimulation, learning, and aging change neural structures and functions. The processes that these factors may induce are morphological alterations in brain areas, changes in neuron morphology, network alterations including changes in neural connectivity, the generation of new neurons (neurogenesis), and neurobiochemical changes. Here we review several aspects of neuroplasticity and discuss the functional implications of the neuroplastic capacities of the adult and differentiated brain with reference to the history of their discovery.” (Fuchs and Fluegge, 2014, p. 1).

It is necessary to bring these research observations regarding steroids, adrenaline, neurotransmitters, nerve growth factors, testosterone and progesterone, ritalin, and vitamin B alimentaries, etc. into focus as well, since Fuchs and Fluegge have emphasized the enormous complexity of “adult neuroplasticity” by likewise pointing out how loose the interactions between endocrinologists, neurophysiologists, geneticists, pharmacologists, ecologists, geriatricians, and neuroanatomists actually are (Mehler, 2008). One major epistemological difficulty to harness and enhance regenerative phenomena for the project of *restorative medicine* is that almost all the contributing disciplinary specialists converse about “regeneration” through their respective, disciplinary “thought styles,” yet mean very different entities by it. Previously Fleck had understood a “thought collective” mostly as determined by its genuine “thought style” driven by an intrinsic “harmony of illusions” (the internal agreement with one preferred working hypothesis—here in neuroscientific thought communities) among the researchers involved:

“After all, conceptions are not logical systems, no matter how much they aspire to that status. They are stylized units which either develop or atrophy just as they are or merge with their proofs into others. Analogously to social structures, every age has its own dominant conceptions as well as remnants of past ones and rudiments of those of the future. It is one of the most important tasks in comparative epistemology to find out how conceptions and hazy ideas pass from one thought style to another, how they emerge as spontaneously generated pre-ideas, and how they are preserved as enduring, rigid structures [*Gebilde*] owing to a kind of harmony of illusions. It is only by such a comparison and investigation of the relevant interrelations that we can begin to understand our own era.” (Fleck, 1979, p. 28).

The same could be said about the objects that “neuroregeneration” or “neuroplasticity” really were, especially when one apprehends the shift from a nineteenth-century thought style about the fixed location of brain functions in the hard-wired morphology of the central nervous system to the acceptance of plastic and adaptive structural processes since the 1960s and 1990s respectively latter half of the 20th century. However, turning Fleck’s assessment here on its head for the sake of the epistemological argument, one could say that the “harmony of illusions” (Ibid., p. 28) between specific disciplinary-bound thought styles really became an “illusion of regeneration harmonies” *vis-à-vis* the existing thought communities from the disciplines of endocrinology to neuroanatomy. In the future, such gaps certainly need to be epistemologically addressed. It is imperative that an integration of the research localities into a functional whole can be reached from a history and philosophy of science perspective, so that new investigative styles of neurophysiological research are becoming possible.

## Discussion

This essay has looked at three areas of “brain regeneration phenomena,” taking primarily morphological research advances into account to highlight some positive as well as negative practical implications of theory dynamics in modern biomedicine. Thereby, the example of “brain regeneration phenomena” since the latter decades of the 19th century displays at least two of Ludwik Fleck’s epistemological structures of theory change—namely the unidimensional “succession of thought styles” and the complex “harmony of illusions.” The period from the 1890s to the early 1960s witnessed the eventual supersession of the “brain rigidity dogma” through the acceptance of a new “plastic regeneration” in the brain thought style (Kandel et al., 1991). In a recent journal article entitled “Ludwik Fleck where are you now that we need you? Covid-19 and the Genesis of Epidemiological Facts,” French historian of science Ilana Loewy has emphasized that “Fleck wished to stimulate the development of the ‘sociology of scientific styles’—a discipline that promotes the understanding of how science works, not as an abstract ideal but as a concrete, situated social practice” (Loewy, 2020, p. 8). In following this vein of analysis, Fleck does hold an epistemological “surprise for us,” *viz.* that more uncertainty had arisen through brain regeneration knowledge from the 1960s to the late 1990s, including work on gene expression, stem cells, LTP-variants, functional mutability, neural connectome complexity, etc., (Frey, 2001), which have led to a deceitful philosophical certainty under the “illusion of regenerative harmony.”

Ludwik Fleck’s concepts and theoretical insights have thus been applied in this essay to the investigation of traditionalist views among groups of neuroscientists that addressed new brain regeneration phenomena in a social context of indetermination and uncertainty. The focus has hereby been laid on questions of the structure and function of scientific concepts of neuroregeneration and neural plasticity, the development of specific “thought communities” of investigators, as well as the impact of new phenomena established through innovative methodologies and laboratory instruments in twentieth-century neuroscientific research endeavors. The resulting “thought communities” of neuroscientists strongly influenced and shaped the acceptance of new concepts about neural plasticity and brain regeneration in the adult human brain.

## Summary and Conclusion

This essay has sought to emphasize the fundamental changes in mostly discipline-bound (as well as certain interdisciplinary) neuromorphological thought styles and epistemologies in modern brain regeneration research. Many neuroanatomists at the beginning of the 20th century had shifted their research focus to the cellular properties of neural de- and regeneration phenomena, a development which laid the basis for a new tradition in the history of neuroplasticity, beginning with neurohistologist Cajal in Spain (DeFelipe and Jones, 1992). These new frontiers in contemporary brain sciences were stimulated by the continuous introduction of newer staining technologies for neurohistology, giving rise to a better understanding of the morphological properties involved in mammalian and human neuroadaptive processes (Bethe, 1895). The gold-derivative staining method applied by Italian neurohistologist Camillo Golgi and the methylene blue dye of German microbiologist Paul Ehrlich (1854–1915) can be named in this respect. Both stains were later used by Bethe (Bethe, 1930) in Frankfurt and also Bielschowsky in Berlin, Germany, within their early research on “neural plasticity” until the 1930s and 1940s.

Such staining techniques also gave rise to continued methodological discussions in contemporary nervous degeneration and regeneration research programs (Pannese, 1996). Modern historians and philosophers of science have since come to use the concept of “emergent functions” to explain such functional hierarchies in more intricate terms (Craver, 2007), while discipline-bound “thought collectives” still exist in recent neuroscientific regeneration research based on protein bioengineering, stem cells, and gene editing (Young, 2009). With Fleck’s insights into the progress and failures of biomedical research, it appears opportune to state that in as much as these thought collectives may trigger normal advances in neuroscience, they apparently also hinder interdisciplinary progress through the provision of an “illusion of regenerative harmony” to a non-negligible degree.
